# Ancestral childhood environmental exposures occurring to the grandparents and great-grandparents of the ALSPAC study children

**DOI:** 10.12688/wellcomeopenres.16257.1

**Published:** 2020-09-04

**Authors:** Jean Golding, Steven Gregory, Sarah Matthews, Daniel Smith, Almudena Suarez-Perez, Claire Bowring, Yasmin Iles Caven, Karen Birmingham, Marcus Pembrey, Matthew Suderman, Kate Northstone

**Affiliations:** 1 Bristol Medical School (PHS), University of Bristol, Bristol, BS8 2BN, UK

**Keywords:** ALSPAC, grandparents, great-grandparents, childhood trauma, transgenerational response

## Abstract

**Background:** Cohort studies tend to be designed to look forward from the time of enrolment of the participants, but there is considerable evidence that the previous generations have a particular relevance not only in the genes that they have passed on, their cultural beliefs and attitudes, but also in the ways in which previous environmental exposures may have had non-genetic impacts, particularly for exposures during fetal life or in childhood.

**Methods:** To investigate such non-genetic inheritance, we have collected information on the childhoods of the ancestors of the cohort of births comprising the original Avon Longitudinal Study of Parents and Children (ALSPAC). The data collected on the study child’s grandparents and great grandparents comprise: (a) countries of birth; (b) years of birth; (c) age at onset of smoking; (d) whether the ancestral mothers smoked during pregnancy; (e) social class of the household; (f) information on 19 potentially traumatic situations in their childhoods such as death of a parent, being taken into care, not having enough to eat, or being in a war situation; (g) causes of death for those ancestors who had died. The ages at which the individual experienced the traumatic situations distinguished between ages <6; 6–11, and 12–16 years. The numbers of ancestors on which data were obtained varied from 1128 paternal great-grandfathers to 4122 maternal great grandmothers. These ancestral data will be available for analysis to *bona fide* researchers on application to the ALSPAC Executive Committee.

## Introduction

A fundamental aim of life-course epidemiology is to understand the determinants of developmental variation in the population and how this relates to health and wellbeing. There is international recognition of the importance of environmental factors such as diet, smoking, social circumstances and stressful events, in influencing child growth, behaviour and neurocognitive development ( Golding *et al*., 2009). In parallel, there is considerable evidence from twin, adoption and family studies that most of these outcomes have a strong familial component ( Golding, 2009). Nevertheless, genome wide association studies of DNA variants often explain little of the heritability of the trait/condition (e.g. Rich, 2016), so other aspects of inheritance need to be considered. 

Growing evidence indicates that the effects of exposures can be transmitted to the next or subsequent generations in some way. These effects are called *intergenerational* if the exposure could have reached the germ cells leading to the next generation(s), or *transgenerational* if this is not the case. The latter implies that some molecular ‘memory’ of the ancestral exposure is being passed down via the gametes; a prime candidate being transgenerational epigenetic inheritance ( Sharma, 2017).

There is strong animal-based experimental evidence for these phenomena, but little to date in humans. In line with animal experiments, there is observational evidence that parental and ancestral early-life experiences contribute to developmental variation in humans, beyond that attributable to ecological and cultural transmission or classic genetic inheritance (reviewed in Pembrey *et al*., 2014). Historical studies from Överkalix in Sweden showed associations between the paternal grandfathers’ food supply in mid-childhood (before the onset of puberty – historically 9–11 years of age) with both longevity and deaths from diabetes in grandchildren ( Bygren *et al*., 2001). Subsequent analysis indicated some sex-specificity in these transgenerational associations such that the paternal grandfather’s food supply was linked to the mortality rate in grandsons but not granddaughters ( Pembrey *et al*., 2006). This has been independently replicated ( Vågerö *et al*., 2018).

In contrast, exposure of the paternal grandmother *prenatally* and in infancy to times of very poor harvests were associated with significantly increased mortality rates of her granddaughters but not her grandsons ( Pembrey *et al*., 2006). Thus, the presumed transmission of these effects is from the *in-utero* exposure of the paternal grandmother to her son and subsequently to his daughter.

A study of exposure in mid-childhood to the German 1916-18 famine looked at economic and related outcomes in later generations. Exposure of the paternal grandfather was associated with better mental health scores in his grandsons. There was also some indication of a similar positive association between the maternal grandmother’s adverse exposure and her granddaughter’s well-being. Exposures at around the age of 9 years were shown to have the greatest effect ( Van Den Berg & Pinger, 2016). The authors suggested that the effects reflected biological responses to adaptive expectations about scarcity in the environment, and as such they could be seen as a correctional mechanism, with marked implications for the offspring. However, several authors have raised the question as to whether effects shown with exposure to famine are actually the consequences of psychological stress, thus complicating the interpretation of which exposures might be inducing intergenerational effects ( Yehuda *et al*., 2008).

When designing the current data collection, the above features in the literature, were considered together with our own studies showing that fathers who started smoking prior to 11 years had offspring who had greater fat mass in late adolescence ( Golding *et al*., 2019), and parents who described their mid-childhood (6–11 years) as less than very happy (an indicator of possible stress) had children who were at increased risk of poor motor coordination ( Golding *et al*., 2014).

**Figure 1.**
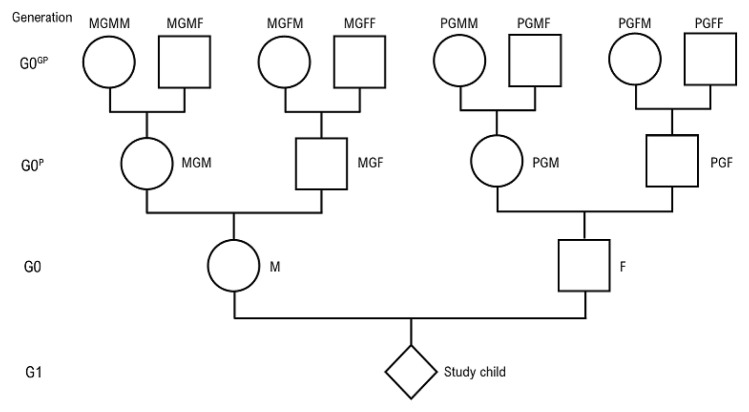
Diagram showing the relationships and nomenclature of the great-grandparents (G0 ^gp^) and grandparents (G0 ^p^) with the parents (G0) and the study child (G1).

The aim of this data collection was to provide information for ourselves and other scientists to identify exposures to the study grandparents and great-grandparents occurring during pregnancy or their childhoods that may have had an inter/trans-generational impact on the study parents and/or their children (see Figure 1 for pictorial depiction of the different routes of inheritance and the nomenclature used in this paper).

## Methods

### Participants

A total of 14,541 pregnant women resident in the former county of Avon in South West England were recruited into the ALSPAC study. These mothers all had an expected delivery date between the 1 ^st^ April 1991 and 31 ^st^ December 1992. From these pregnancies, there were a total of 14,676 fetuses and 14,062 live births. Of these children, 13,988 were still alive at 1 year of age. Mothers were considered enrolled if they had returned at least one questionnaire or attended a “Children in Focus” clinic by 19 ^th^ July 1999. At the age of 7, the study team reached out to mothers who had previously not been included in the study and recruited additional eligible families in order to boost the number of participants. As such, from the age of 7 the total sample number is 15,454 pregnancies, resulting in 15,589 fetuses, of which 14,901 were alive at 1 year of age ( Boyd *et al*., 2013; Fraser *et al*., 2013; Northstone *et al*., 2019). In order to protect the confidentiality of the sample, data from triplet and quadruplet pregnancies have been removed as these children were considered to be at risk of identification. ALSPAC is continuing to monitor all families in the study and are recruiting the Children of the Children of the 90s ( Lawlor *et al*., 2019).

Following the advice of the ALSPAC Ethics and Law Committee, partners were originally recruited into the study only if the enrolled mothers wished them to be included. Questionnaires were sent to the mother who then passed the questionnaire on to the partner with a separate pre-paid return envelope. This method meant that ALSPAC were unable to follow up or communicate directly with the partners ( Birmingham, 2018). Therefore, the numbers of partners’ questionnaires returned were less than those received for the mother’s questionnaires. In all, around 75% of the partners participated in the study at some stage.

### The nomenclature used here

 For the past 10 years the parents of the study children have been known as the G0s and their offspring, the actual Children of the Nineties, as the G1s (G representing generation). The subsequent births (Children of the Children of the Nineties or CoCo90s) have been referred to as the G2s. This works well. However, when referring back to ancestors it has often been found confusing and sometimes ambiguous, to refer to these as the G1s or G2s. We therefore have changed the nomenclature when discussing these ancestors, and will henceforth use G0 ^p^ to denote the parents of the G0 population (i.e. the grandparents of the G1s), and G0 ^gp^ for the grandparents of the G0s (i.e. the great grandparents of the G1s) ( Figure 1).

### The ancestral questionnaires

**Figure 2.**
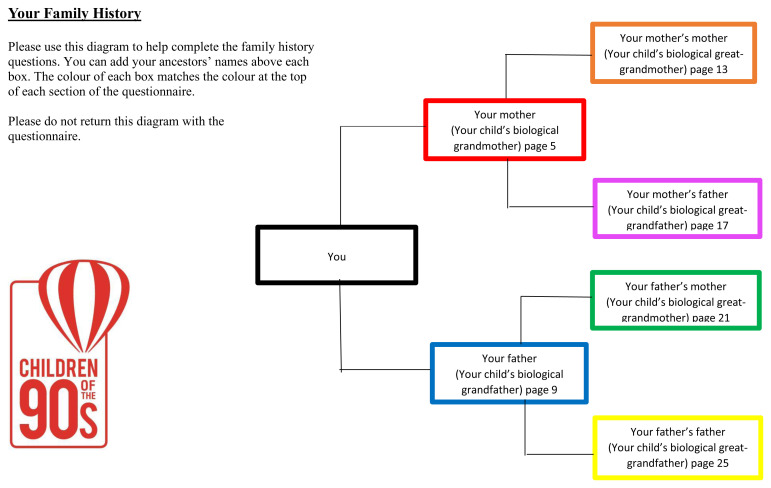
The form sent with the questionnaire for the parent to fill in and use as an aide memoire.

Questionnaires were designed to ascertain information from the study mothers and (the presumed biological) fathers [G0] concerning each of six relatives: their two parents (the study child’s grandparents) [G0 ^p^] and their four grandparents (the study child’s great grandparents) [G0 ^gp^]. To avoid confusion, a family tree was provided, with each ancestor allocated a different colour. For example, see Figure 2 - the family tree for study mothers; a similar tree but with different colours was provided for the study fathers. Each parent was invited to complete the tree for their own use with the names of each ancestor. Each set of questions was outlined with the relevant background colour for that relative (Extended data: Family History Questionnaire; Iles Caven *et al*., 2020). The nomenclature used in the questionnaires for each individual in the family tree is indicated in Box 1.

**Box 1. Questionnaire nomenclature**

**Table:** 

	*Maternal line*
M	Mother
MGM	Maternal grandmother
MGMM	Maternal grandmother’s mother
MGMF	Maternal grandmother’s father
MGF	Maternal grandfather
MGFM	Maternal grandfather’s mother
MGFF	Maternal grandfather’s father
	*Paternal line*
F	Father
PGM	Paternal grandmother
PGMM	Paternal grandmother’s mother
PGMF	Paternal grandmother’s father
PGF	Paternal grandfather
PGFM	Paternal grandfather’s mother
PGFF	Paternal grandfather’s father

The initial question to the respondent established the study participant’s relationship to the study child [G1]. If they were known not to be the biological parent, they were asked to complete the questions for the study child’s biological ancestors, if possible.

Further questions about each of the ancestors included their date of birth and place of birth (i.e. country and town or village); if they moved during childhood and if so, where to and at what age; if they were still alive and if not, their age at death or date of death, place of death and cause of death. They were also asked about each of their ancestors’ occupation(s); number and gender of their siblings; if siblings were older, younger or a twin and if so, identical or non-identical; if they smoked during childhood and if so, at what age they started; for female ancestors only, whether they smoked when pregnant with the study child’s direct ancestor (i.e. with the study child’s mother or father [G0] or grandparents [G0 ^p^]).

 The potentially traumatic situations concerned whether the ancestor had: suffered from a serious illness; attended boarding school; been taken into care by family or others; had been in a war situation; became a refugee; had been subjected to violence, directly or whether there was violence in their home; not enough to eat at times or had an unhappy childhood. In addition, during their childhood whether any of the following had occurred to their parents including whether either had died, been seriously ill, been in a war situation or become a refugee. Finally, they were asked to describe any other major events or additional comments concerning their ancestor’s childhood. The questionnaires were approved by the ALSPAC Ethics and Law Committee on 26 ^th^ February 2018 (Ref 60602).

### Distributing the questionnaires

The questionnaire was available to complete in either online or paper format. Participants were not contacted if our administrative database record indicated that they were deceased, had withdrawn from the study, had declined further contact or had declined to complete questionnaires.

The questionnaire was sent to 9149 mothers and 3230 enrolled fathers [G0] (n= 12,379). Where the mother did not have a linked enrolled father on the database, they were asked if they were happy to send a questionnaire on to their partner to complete about his ancestors. In all 411 mothers requested a paper questionnaire to be sent to them to pass on to the non-enrolled study fathers, 405 were actually sent out.

**Figure 3.**
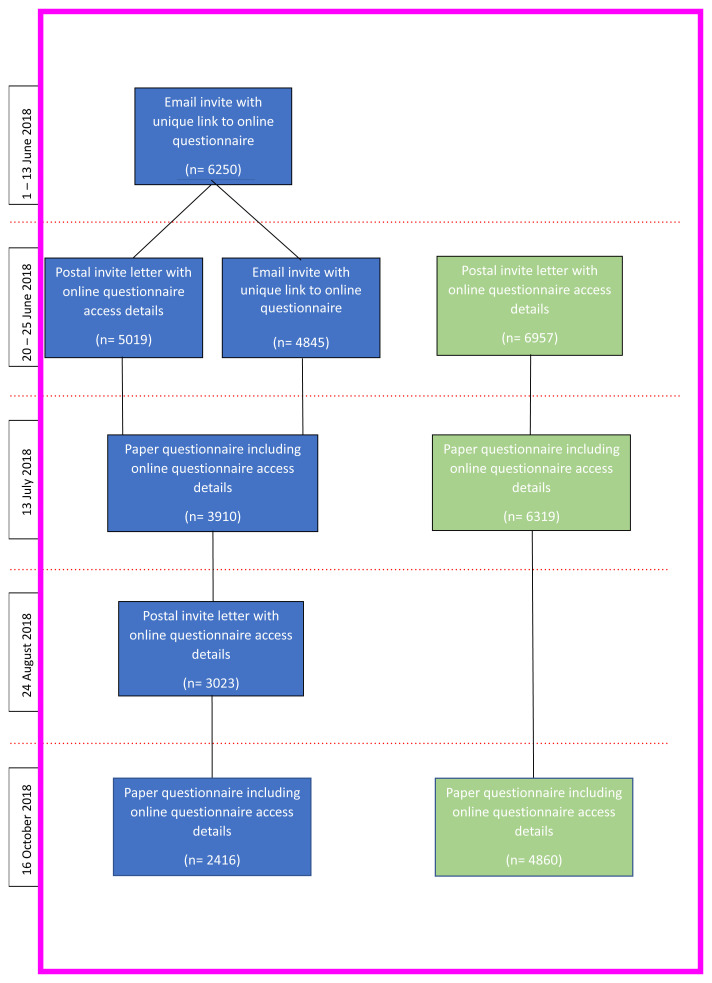
Flow diagram concerning the invitations and reminders sent to the study parents (G0s).

The G0 participants with an email address were sent an initial email invite (with an online questionnaire link) at the beginning of June 2018, followed by a series of reminder emails, letters and paper copies of the questionnaire to participants who had not responded. G0 participants without an email address were sent an initial letter of invitation followed by two paper copies of the questionnaire to those who had not responded ( Figure 3).

Participants received a £10 shopping voucher for completing the questionnaire and, provided they were happy for this to happen, they were entered into a prize draw to win one of three iPads.

Completed paper questionnaires were scanned into electronic data using Teleform data capture software. Data collection for the online questionnaires were collected and managed using REDCap (electronic data capture tools hosted at the University of Bristol ( Harris *et al*., 2009; Harris *et al*., 2019)).

### Coding the questionnaires

For questions concerning moving during childhood and traumatic childhood experiences (questions 2 and 3 within each section), there were potential pitfalls and complications. Each such question (Appendix 1) allowed six different responses: four positive (yes <6, yes 6–11, yes 12–16 and yes, age not known), one negative (did not happen) and one don’t know (unknown if happened or not). The setup of REDCap meant that six binary variables were generated for each of these questions. Given the strategy of labelling the binary variables yes/no, a positive response may indicate a negative event (e.g., answering “yes” to “maternal grandmother did not move during childhood”, to indicate not moving). For these questions there was also a “don’t know” option; this means that a lack of response to “maternal grandmother did not move during childhood” does not mean that they did move during childhood, as the respondent could have answered “don’t know” to this other question. To help improve clarity for researchers using these data, for each question the first four variables have been put to missing if ‘’don’t know’ was ticked. A new variable was derived from these six responses to identify those who had experienced the event at any age (including age NK) and those known not to have had such a history. 

Much of the text data from this questionnaire has been coded into numeric variables in order to be readily accessed by researchers. These include: country of birth (based on ISO 3166-1 codes https://www.iso.org/obp/ui/#search), and stated cause of death using self-generated codes. Social class data has been derived from occupational text which was coded using Computer Assisted Structured Coding Tool (CASCOT). This software uses the UK standards developed by the Office for National Statistics and is available from the Warwick Institute for Employment Research using UK SOC 2010 v.7 ( https://warwick.ac.uk/fac/soc/ier/software/cascot/).

### Ethical approval and consent

Prior to commencement of the study, approval was sought from the ALSPAC Ethics and Law Committee and the Local Research Ethics Committees ( Birmingham, 2018). Informed consent for the use of data collected via questionnaires and clinics was obtained from participants following the recommendations of the ALSPAC Ethics and Law Committee at the time. Questionnaires were completed in the participants own home and return of the questionnaires was taken as continued consent for their data to be included in the study. Full details of the approvals obtained are available from the study website ( http://www.bristol.ac.uk/alspac/researchers/research-ethics/). Study members have the right to withdraw their consent for elements of the study or from the study entirely at any time.

## The data collected

Overall, 4660 and 2182 completed questionnaires were received from the study mothers and fathers respectively. Of these, 65% and 66% were completed online, and the remainder on paper. Only 0.1% and 4.1% of those who replied stated they were not the biological parent – they almost entirely described themselves as step-parents. They were asked to reply with the information relevant to the biological parent. The numbers of details received varied with the relationship, with about twice as many sets of information related to the maternal line compared with the paternal line (see Column A in Table 1). Not surprisingly, more was known about the grandparents than the great-grandparents. Nevertheless, data were provided for over 1100 ancestors in the paternal line and over 2100 ancestors in the maternal line.

**Table 1.** Features of the grandparents and great-grandparents.

Ancestor	A	B	C	D %	E %	F %	G %	H %
**MGM**	4122	1905-1962	1934	16.1	17.9	25.0	12.8	10.6
**MGF**	3981	1898-1959	1931	16.6	45.8	-	12.8	11.1
**MGMM**	3116	1866-1940	1904	19.3	17.2	14.1	6.6	26.2
**MGMF**	2685	1810-1940	1901	19.2	60.5	-	7.5	24.6
**MGFM**	2497	1859-1944	1901	19.9	16.1	12.2	7.6	24.8
**MGFF**	2224	1803-1940	1899	18.9	64.9	-	7.4	27.1
**PGM**	2182	1899-1952	1930	17.4	17.1	22.8	13.1	11.1
**PGF**	1900	1878-1950	1927	17.8	42.1	-	12.3	11.4
**PGMM**	1363	1858-1940	1900	21.0	16.2	11.6	8.7	26.1
**PGMF**	1231	1860-1930	1898	20.3	60.2	-	7.6	22.5
**PGFM**	1173	1860-1927	1898	19.6	15.2	10.1	7.3	25.4
**PGFF**	1128	1850-1938	1896	18.9	60.5	-	7.0	24.5

A=Numbers of individuals for whom information was available; B = range of years of birth; C = median year of birth; D = proportion [n] born outside of England; E = proportion [n] who had started to smoke in childhood; F = proportion of mothers who had smoked during the pregnancy; G = proportion who were ‘only’ children, and H = proportion with more than five siblings.

### Demographic data

 Information on the years of birth of each of the 12 ancestors are shown in Columns B and C of Table 1. The median year of birth of the maternal and paternal grandparents was in the period 1927–1934 – i.e. after the First World War and before the Second. On average, the paternal grandparents were born about three years before the maternal grandparents both between genders overall and within pairs of grandparents. A similar pattern was shown for the great-grandparents. For all the male ancestors there was a wide range in their years of birth; the majority of great grandfathers were born before the First World War.

 The proportion of each group of ancestors born outside England is shown in Column D of Table 1. This shows that 16–17% of grandparents and slightly more great-grandparents (19–21%) were born outside of England. This mainly included the rest of the British Isles and countries that were then in the British Empire.

 The number of younger and older brothers and sisters were ascertained for each ancestor. The size of the tails of the distribution of the total numbers of siblings (which ranged from 0 to 24) are shown in Columns G and H of Table 1. In general, 12–13% of grandparents were ‘only’ children, but fewer great-grandparents (7–8%) were in this category. At the other end of the distribution, about 11% of grandparents had 6 or more siblings, but this was true of 24–27% of the great-grandparents.

### Smoking

The questions on smoking concerned smoking in childhood (i.e. <17) and, for the female ancestors, whether they had smoked when pregnant with the next in line. Thus, for the MGM or PGM groups, this would refer to whether they smoked when pregnant with the study mother or father respectively; For the great-grandmothers, whether they smoked in the pregnancy resulting in the birth of the grandparent. The numbers answering each question are shown in columns E and F of Table 1. There is a large difference in the onset of regular smoking in childhood between the male and female ancestors. For the great-grandfathers about 60% had started smoking in childhood in comparison with about 16% of great-grandmothers. However, more of the great-grandmothers were smoking at the time of pregnancy (23–25%). For the actual age at which the male and female ancestors had started to smoke regularly, few had reported doing so before 11 years of age, and most reported that this habit had started when they were aged 14 (the earliest school leaving age, and probably the age at which they started work or an apprenticeship). 

### Causes of death

 The causes of death of those ancestors who had died were written as text, and subsequently coded. Separate codes were created for 34 types of condition. These have been condensed into the eight groups shown in Table 2. This shows that there were numerically more deaths among the male ancestors (MGF, MGMF, MGFF, PGF, PGMF, PGFF) than their female counterparts (MGM, MGMM, MGFM, PGM, PGMM, PGFM) for lung problems and deaths associated with violence (Columns C and D), whereas the female ancestors were more likely to be reported as dying with dementia and with multiple problems including old age (Columns A and B). It must be remembered, however, that many of the G0 ancestors were still alive at the time the questionnaires were completed (2019).

**Table 2.** Numbers of ancestors for whom causes of death have been given, and the numbers that were still alive in autumn 2018.

Ancestor	A	B	C	D	E	F	G	H	All Known ^a^	% Alive ^b^
**MGM**	122	171	73	35	373	177	622	333	1655	57.5
**MGF**	104	137	133	78	773	244	884	423	2429	35.0
**MGMM**	347	142	57	59	728	224	518	363	2201	1.2
**MGMF**	128	48	137	92	696	184	468	277	1881	<0.5
**MGFM**	254	101	44	45	502	147	356	246	1544	0.8
**MGFF**	95	20	91	80	504	161	348	200	1376	<0.5
**PGM**	109	101	32	29	222	96	323	194	985	44.7
**PGF**	82	62	69	40	415	114	439	270	1313	25.9
**PGMM**	172	45	16	19	205	69	196	129	774	0.8
**PGMF**	62	14	45	41	257	67	196	103	724	<0.7
**PGFM**	135	42	8	24	128	50	146	107	591	<0.9
**PGFF**	64	13	56	49	202	67	139	98	640	<0.8

Causes of death: A = multiple problems / old age; B = Dementia; C = Lung problems/COPD; D = Accident/violence/suicide; E = Cardiovascular; F = Infections; G = Cancer; H = Miscellaneous causes. ^a^No. with known causes of death; ^b^Percentage of the total numbers in Column A of Table 1

### Potentially traumatic situations in childhood

 As shown in Appendix 1, the questionnaire enquired about 19 different situations that the ancestor may have experienced during their childhoods. For each situation, the age at which the situation occurred was asked, with the following possible options: < 6 years; 6–11 years; 12–16 years; occurred in childhood but age not known. The numbers experiencing such situations at any age are shown for the maternal and paternal ancestors in Table 3. Not surprisingly, given the years in which they were born, the most common situations concerned either themselves or their parents being in a war. The next most common situation reported was that there was not enough to eat. Relatively few reported being refugees, but other frequent traumas included a parent being seriously ill or dying, being subjected to violence or being taken into care.

**Table 3.** The numbers of the mothers’ (A) and fathers’ (B) ancestors who reported having experienced potentially traumatic situations in childhood. For each situation, the age group at which it occurred is available.

Situation in childhood	MGM	MGMM	MGMF	MGF	MGFM	MGFF
**(A) Mothers’ ancetors**						
Seriously ill	745	169	122	483	77	63
Boarding school	252	67	83	306	27	61
Taken into care by family	176	105	60	192	64	45
Taken into care – other	194	73	44	147	44	30
In war situation	2602	1521	1181	2474	988	828
Refugee	75	26	18	91	15	11
Subjected to violence	163	57	69	229	21	57
In a violent household	190	69	50	225	32	46
Not enough to eat	811	534	370	755	297	237
Was unhappy	711	156	101	460	97	67
Her/his mother died	401	300	214	382	207	123
Her/his mother was seriously ill	541	214	127	355	135	62
Her/his mother was in a war situation	2519	1036	721	2236	607	476
Her/his mother was a refugee	48	17	11	34	15	7
Her/his father died	619	317	214	632	204	140
Her/his father was seriously ill	521	161	87	451	97	49
Her/his father was in a war situation	2414	970	684	2200	565	459
Her/ his father was a refugee	36	18	11	31	13	8
Other trauma (described)	452	148	109	310	110	79
**(B) Fathers’ ancestors**						
Seriously ill	224	59	37	190	23	20
Boarding school	111	18	35	133	13	42
Taken into care by family	98	37	25	84	21	15
Taken into care – other	81	15	14	57	19	17
In war situation	1207	532	416	1133	354	319
Refugee	30	8	7	34	5	<5
Subjected to violence	50	14	20	66	6	17
In a violent household	55	14	17	59	<5	14
Not enough to eat	344	177	119	324	119	99
Was unhappy	200	27	30	153	17	16
Her/his mother died	228	87	70	235	73	54
Her/his mother was seriously ill	189	55	29	165	33	22
Her/his mother was in a war situation	1163	316	244	1029	224	172
Her/his mother was a refugee	21	6	<5	25	5	<5
Her/his father died	314	103	76	319	73	76
Her/his father was seriously ill	217	42	33	201	26	29
Her/his father was in a war situation	1093	298	223	1012	209	166
Her/ his father was a refugee	18	5	7	19	<5	<5
Other trauma (described)	157	37	33	140	29	30

### The variable nomenclature

 There are a large number of variables created for this project. The variable numbering system is indicated in Table 4. 

**Table 4.** The structure of the variable labels for the 12 different ancestors.

Question no. ^a^	Variable ^b^	Description
G0	W *000_M	Mother able to answer questions about this ancestor
G1	W *010_M	Year of birth
G1a	W *011_M	Born in England
DV	W *015_M	Place/country of birth
G2	W *022_M W *023_M W *024_M W *025_M W *026_M	Moved area in childhood
G3a	W *030_M W *031_M W *032_M W *033_M W *036_M	Seriously ill in childhood
G3b	W *040_M W *041_M W *042_M W *043_M W *046_M	Went to boarding school
G3c….G3r	….	……
G3s	W *210_M W *211_M W *212_M W *213_M W *216_M	Other major event in childhood
G4	W *220_M	Smoked regularly in childhood
G4a	W *221_M	Age started smoking
G5	W *230_M	No. of siblings
G5a	W *231_M	No. younger brothers
G5b	W *232_M	No. younger sisters
G5c	W *233_M	No. older brothers
G5d	W *234_M	No. older sisters
G5e	W *235_M	Whether a twin
G5f	W *236_M	Whether an identical twin
G7	W *251_M	Social Class (based on occupation)
G8	W *260_M	Whether still alive
G8a	W *261_M	Age at death
G8bmm	W *263_M	Month of death
G8byyyy	W *262_M	Year of death

^*^This should be substituted by the following numbers, depending on the ancestor: MGM/PGM = 2; MGF/PGF=3; MGMM/PGMM=4; MGMF/PGMF=5; MGFM/PGFM=6; MGFF/PGFF=7. ^a^The question number is preceded by a different letter for each ancestor as shown in the Questionnaire in Appendix 1: MGM/PGM = B; MGF/PGF=C; MGMM/PGMM=D; MGMF/PGMF=E; MGFM/PGFM=F; MGFF/PGFF=G. ^b^The paternal line is as above but instead of ‘_M’ insert ‘_F’

## Discussion

 As far as we are aware, this is the first cohort study to have attempted to obtain information on the childhoods of the ancestors of the study cohort. As will have been seen, there are many gaps in the information collected on grandparents and especially great-grandparents in the study. Nevertheless, there are a number of instances where there are sufficient numbers available, and therefore sufficient statistical power, for analysis of possible consequences to the G1 generation when using continuous variables that may be available on them.

It is crucial that there is some evaluation of the validity of the data themselves. There are indirect signs of validity in that the rate of smoking of the ancestors was much higher among the men than the women, with reported onset of smoking at age 14, which was the school leaving age, and will have been the age at which they will most likely have started work. These reports reflect accurately what is known about smoking in Britain in the first half of the twentieth century ( Forey *et al*., 2016)

 We have therefore carried out a validity study whereby the questionnaire results are compared with those from in-depth interviews with the participating parent. The results will be the subject of a separate data note. In addition, we are able to compare the results of data collected from both mothers and fathers on the study grandmothers’ prenatal smoking habits during pregnancy with the results in this survey, 27–28 years later. The results are particularly gratifying in that there was good test-retest reliability (kappa = 0.44 for mothers; 0.84 for fathers).

One of the intriguing aspects of this data collection is the evidence of the frequency of potentially stressful situations experienced by these ancestors, particularly in regard to aspects such as exposure to war, domestic violence and other traumatic events (described in more detail by Birmingham *et al*., 2020 under review). The most common event recorded in this study was exposure to war during childhood. Although it is assumed that such an event is traumatic, there is much evidence that many children, particularly boys, thrived during the war - they enjoyed watching dog fights overhead and exploring bombsights for pieces of shrapnel, which they traded with one another (e.g. Courtenay, 2000). It is only in exceptional circumstances that the exposure to the Second World War in Britain exposed children to extreme deprivation. Nevertheless, in this study we have shown that there were considerable numbers of ancestors described as experiencing being hungry as well as being exposed to violence of various sorts. The identity of the various events that we have documented that might have longitudinal consequences on the subsequent generation(s) is available for exploration.

Although the aim of the principle investigators (JG and MP) was to use the data to look at transgenerational effects of exposures in preceding generations, there are many other research questions that can be addressed by these data.

### Strengths and limitations

The major strength of this data set is that, to our knowledge, it is unique. Although some data linkage of records in the Scandinavian and other countries may be able to examine certain aspects of transgenerational effects, there has been no systematic collection of evidence of potentially traumatic environmental effects occurring in childhood. The fact that these data were collected from a geographically based population of individuals, unselected by aspects such as health or education, provides an added advantage, as does the wealth of data available on the G0 and G1 generations to which these historical reports are linked.

There are limitations to the study, however. Firstly, we show that there is often incomplete knowledge from the study participants as to the childhoods of their ancestors. Secondly, although we have tested validity using a test-retest paradigm, this does not compare with a gold standard. Thirdly, for comparative purposes there are rarely any studies with which any results may be directly compared.

## Conclusions

There are many reasons why it may be important to determine whether ancestral exposures may have a detectable effect on the outcomes of future generations. There have been few studies aimed at making such determinations. By collecting the information described here on the great-grandparents (G0 ^gp^) and grandparents (G0 ^p^) of the children (G0) taking part in the ALSPAC cohort, and linking such data to the wealth of information collected on them and their study parents (G0) and their own children (G2), the potential to look intergenerationally and trans-generationally at ancestral fetal and childhood exposures is available. To our knowledge this is the first birth cohort study to have collected information on five generations of the same family.

## Data availability

### Underlying data

ALSPAC data access is through a system of managed open access. The steps below highlight how to apply for access to the data included in this data note (project B2362) and all other ALSPAC data:

1. Please read the ALSPAC access policy (PDF, 844kB) which describes the process of accessing the data and samples in detail, and outlines the costs associated with doing so.
2. You may also find it useful to browse our fully searchable research proposals database, which lists all research projects that have been approved since April 2011.
3. Please submit your research proposal for consideration by the ALSPAC Executive Committee. You will receive a response within 10 working days to advise you whether your proposal has been approved.

### Extended data

Figshare: Ancestral childhood environmental exposures occurring to the grandparents and great-grandparents of the ALSPAC study children: Family History Questionnaire. https://doi.org/10.6084/m9.figshare.12866597 ( Iles Caven *et al*., 2020).

This project contains the family history questionnaire used to generate the data described in this note.

Extended data are available under the terms of the Creative Commons Attribution 4.0 International license (CC-BY 4.0).
