# Supplementary material for: Ancestral childhood environmental exposures occurring to the grandparents and great-grandparents of the ALSPAC study children
Source: Wellcome Open Res. 2020 Sep 4;5:207. [Version 1] doi: 10.12688/wellcomeopenres.16257.1 (PMC7527864; doi:10.12688/wellcomeopenres.16257.1)
Supplement: Peer review history

# Peer review: Reviewer response for version 1

**DOI:** 10.21956/wellcomeopenres.17859.r40304

**Published:** 2020-09-30

**Authors:** Peter Elias

**Affiliations:** 1 University of Warwick, Coventry, UK

This article describes a unique addition to the ALSPAC study, already recognised internationally as a major longitudinal study of parents and children. It describes in some detail the processes of collecting, codifying and integrating information about the maternal and paternal grandparents and great grandparents of study members. The new information added to the study includes the geographical origins of their ancestors, their years of birth and death, smoking during pregnancy for ancestral mothers, the social background of households and information on potentially traumatic events in their childhood.

 As with all epidemiological studies, analysis of the information generated by this recent survey of study members may give rise to some interesting correlations – indicative of, but not confirming, causal influences. The difference with the ALSPAC study lies with the richness of the data already collected, which will allow for control of many confounding factors in the search for intergenerational and, more importantly, transgenerational influences on the physical and mental well being of study members and their children. 

 While the efforts to collect such historical detail within the context of a major longitudinal study are to be lauded, the accuracy and completeness of information passed down as family history should be a major concern. Recognising this as a potential weakness, the study authors describe a test/retest measure of reliability (study grandmothers’ prenatal smoking habits during pregnancy with the results in this survey, 27–28 years later) which shows good reliability. While this is useful, it does not address the fact that study members provided information on the cause of death for only two thirds of maternal grandmothers and fathers, and just over half of paternal grandmothers and fathers. Given the existence of extensive family history records online and open for public search, it would be interesting to see how the cause and date of death records within this study can be extended and validated.

 Despite this weakness, this is a pathbreaking addition to the UK’s collection of birth cohort studies which is likely to stimulate further interest in the mechanisms associated with transgenerational epigenetic inheritance. As the study PI states: ‘To our knowledge this is the first birth cohort study to have collected information on five generations of the same family.’ The development of this research resource represents an important milestone in research efforts to enhance our understanding of cross generational developmental influences. It is also one which will benefit greatly from future enhancements.

Are sufficient details of methods and materials provided to allow replication by others?

Yes

Is the rationale for creating the dataset(s) clearly described?

Yes

Are the datasets clearly presented in a useable and accessible format?

Yes

Are the protocols appropriate and is the work technically sound?

Yes

Reviewer Expertise:

Labour economics, birth cohort studies, longitudinal data resources, classifications, skills and productivity.

I confirm that I have read this submission and believe that I have an appropriate level of expertise to confirm that it is of an acceptable scientific standard.

# Peer review: Reviewer response for version 1

**DOI:** 10.21956/wellcomeopenres.17859.r40305

**Published:** 2020-09-28

**Authors:** Richard E. Tremblay, Massimiliano Orri

**Affiliations:** 1 University of Montreal, Montreal, Canada; 2 McGill University, Montreal, QC, Canada

The number of longitudinal studies on children’s development exponentially increased since Charles Darwin’s pioneering observations of his children’s development in the first half of the 19th century (Tremblay, in press) ^1^. The Golding *et al. *(2020) ^2^ paper on ‘Ancestral childhood environmental exposures …’ using retrospective data from the Avon Longitudinal Study of Parents and Children (ALSPAC), helps to appreciate the extent to which the science of child development has progressed since the middle of the 19th century. 

 The paper describes retrospective information collected on the parents, grandparents, and great-grandparents of the ALSPAC children born during the 1990s. To help the reader appreciate the relatively long historical period involved, it is useful to note that the data was collected in 2018 and includes information on at least one maternal grandfather’s father who was born in 1803. This was 6 years before Charles Darwin’s birth! One maternal grandmother’s father was also born one year after Charles Darwin (1810). To our knowledge, these data provide the first opportunity to study the associations between the life-span developmental trajectories of today’s young adults and the life-span developmental trajectories of their ancestors over two centuries.

 The data on ancestors includes the date of birth and death, causes of death, information on siblings, smoking, potentially traumatic situations in childhood, such as illnesses, being taken into care, being a victim of violence, and attending boarding school. 

 One important limit of the data is that the information obtained from the maternal line of ancestors was much more extensive than the information obtained from the paternal line. This was to be expected since all studies of child development asking for parent’s collaboration obtain much better collaboration from mothers than from fathers. 

 The paper by Golding *et al.* (2020) illustrates very well how the data can be used to study intergenerational effects. We can expect that numerous investigators will be interested in using these retrospective data to complement the high quality, extensive prospective data that was collected on the children born in the 1990s, as well as the data that should be collected as the targeted children, become parents, grand-parents and great grand-parents themselves. ALSPAC is likely to be the most important intergenerational longitudinal study of child development ever, when the 300th anniversary of Charles Darwin’s birth will be celebrated! 

 One of the very interesting questions that the ancestral data can address is the intergenerational consequences of parents’ age at the conception of their children. As highlighted above, there can be extremes in the age of parents at conception. Because of different biological constraints for men and women, this variability is greater for fathers than for mothers. However, there is also room for much age variation among mothers between puberty and menopause (Gao *et al.*, 2019; Marasco, Boner, Griffiths, Heidinger, & Monaghan, 2019). ^3^^,^^4^ The age differences within couples at conception may also substantially change from one generation to another. For example, the great-grandfather of a child’s mother may have conceived the grandfather of the child’s mother when he was in his 60’s, while his son, the grandfather of the child’s mother, may have conceived the father of the child’s mother when he was in his 20’s. 

 The identification and classification of the intergenerational combinations of couples’ age at conception will provide an interesting intergenerational developmental grid which could be an important predictor of the ALSPAC children’s developmental trajectories for various bio-psycho-social developmental dimensions.   

 Indeed, parent’s age at conception may have impacts on a variety of psycho-social outcomes, including wealth, parenting style, and cultural values. But parents' age at conception also impacts biological development. For example, there is evidence that children conceived when their parents were both relatively old reduces the child’s longevity (Eisenberg & Kuzawa, 2018). ^5^ As suggested in the Golding *et al.* paper, there is also good evidence of epigenetic effects (Pembrey, Saffery, & Bygren, 2014) ^6^ associated with environmental life conditions of the parents, and these conditions can be associated to the age of the parent’s at conception. The intergenerational impact of family size could also be examined (Beaujouan & Solaz, 2019; Grinde & Tambs, 2016), ^7^^,^^8^ as well as that of birth rank, if data were collected on the birth date of all siblings within each family (Knodel & Hermalin, 1984; Kristensen & Bjerkedal, 2007). ^9^^,^^10^  

 Golding *et al. *(2020) clearly state that the intergenerational data collection was driven by the human and animal evidence that “early life experiences contribute to developmental variation, beyond that attributable to ecological and cultural transmission or to classic genetic inheritance” (Pembrey *et al.*, 2014) ^6^. With the ancestral data, they collected it is now possible to address these issues for numerous developmental characteristics related to social behavior, educational achievement, illnesses, mate choice, parenting, and longevity. 

 We must be great-full to Jean Golding and her colleagues for having created ALSPAC, but also for continuing to add intergenerational developmental layers of information that will be highly useful to advance research over the next century and more!

Are sufficient details of methods and materials provided to allow replication by others?

Yes

Is the rationale for creating the dataset(s) clearly described?

Yes

Are the datasets clearly presented in a useable and accessible format?

Yes

Are the protocols appropriate and is the work technically sound?

Yes

Reviewer Expertise:

NA

We confirm that we have read this submission and believe that we have an appropriate level of expertise to confirm that it is of an acceptable scientific standard.

## References

[1] : The Science of Violent Behavior Development and Prevention: Contributions of the Second World War Generation. *Cambridge University Press*.2020;

[2] : Ancestral childhood environmental exposures occurring to the grandparents and great-grandparents of the ALSPAC study children. *Wellcome Open Research*.2020;5: 10.12688/wellcomeopenres.16257.1 10.12688/wellcomeopenres.16257.1 33043146PMC7527864

[3] : Overlooked roles of DNA damage and maternal age in generating human germline mutations. *Proceedings of the National Academy of Sciences*.2019;116(19) : 10.1073/pnas.1901259116 9491-9500 10.1073/pnas.1901259116 PMC651103331019089

[4] : Intergenerational effects on offspring telomere length: interactions among maternal age, stress exposure and offspring sex. *Proceedings of the Royal Society B: Biological Sciences*.2019;286(1912) : 10.1098/rspb.2019.1845 10.1098/rspb.2019.1845 PMC679076231575358

[5] : The paternal age at conception effect on offspring telomere length: mechanistic, comparative and adaptive perspectives. *Philosophical Transactions of the Royal Society B: Biological Sciences*.2018;373(1741) : 10.1098/rstb.2016.0442 10.1098/rstb.2016.0442 PMC578406229335366

[6] : Human transgenerational responses to early-life experience: potential impact on development, health and biomedical research. *J Med Genet*.2014;51(9) : 10.1136/jmedgenet-2014-102577 563-72 10.1136/jmedgenet-2014-102577 25062846PMC4157403

[7] : Is the Family Size of Parents and Children Still Related? Revisiting the Cross-Generational Relationship Over the Last Century. *Demography*.56(2) : 10.1007/s13524-019-00767-5 595-619 10.1007/s13524-019-00767-5 30868472PMC6449311

[8] : Effect of household size on mental problems in children: results from the Norwegian Mother and Child Cohort study. *BMC Psychol*.2016;4(1) : 10.1186/s40359-016-0136-1 31 10.1186/s40359-016-0136-1 27255744PMC4890284

[9] : Effects of birth rank, maternal age, birth interval, and sibship size on infant and child mortality: evidence from 18th and 19th century reproductive histories. *Am J Public Health*.1984;74(10) : 10.2105/ajph.74.10.1098 1098-106 10.2105/ajph.74.10.1098 6383084PMC1651865

[10] : Explaining the relation between birth order and intelligence. *Science*.2007;316(5832) : 10.1126/science.1141493 1717 10.1126/science.1141493 17588924
